# Age-Related Changes in the Behavior of Apolipoprotein E Knockout Mice

**DOI:** 10.3390/bs8030033

**Published:** 2018-03-03

**Authors:** Dasha Fuentes, Nidia Fernández, Yenela García, Teidy García, Ana Ruth Morales, Roberto Menéndez

**Affiliations:** 1 National Center for Laboratory Animal Breeding (CENPALAB), Calle 3ra N° 40759 entre 6ta y Carretera Tirabeque, Reparto La Unión, Boyeros, La Habana 10800, Cuba; nidia.fernandez@cenpalab.cu; 2 National Center for Bioproducts (BIOCEN), Carretera de Beltrán, Km. 1 1/2, Bejucal 32600, Mayabeque, Cuba; yenela@biocen.cu; 3 Institute for Marine Sciences (ICIMAR), Calle Loma entre 35 y 37, Plaza de la Revolución, La Habana 10400, Cuba; teidy@cebimar.edu.cu (T.G.); anar@cebimar.edu.cu (A.R.M.); 4 Center for Neurosciences (CNEURO), Ave. 25 y 158 No. 15202, Cubanacán, Playa, La Habana 11300, Cuba; roberto.menendez@cneuro.edu.cu

**Keywords:** aging, ApoE, behavior, mice, neurodegenerative disorder

## Abstract

The knockout mouse model, B6.129P2-Apoe^tm1Unc^ is homozygotic for the Apolipoprotein E (ApoE) deletion; thus, it is capable of developing hyperlipidemia and atherosclerosis but ApoE is also a lipid-transport protein abundantly expressed in most neurons in the central nervous system, so these animals could also be models of neurodegenerative diseases. The aim of this study was to determine age-related changes in spontaneous behavior and in learning and memory of Apolipoprotein E knockout mice. Spontaneous behavioral measurements included sleeping pattern, motor coordination and balance by rotarod and open field activity, whereas learning and memory tests included forced alternation in Y-maze, novel object recognition and passive avoidance conditioning. Significant behavioral differences between aged knockout mice and age-matched wild type strain, C57Bl/6 were found in all the behavioral tests, except for the rotarod test. Genetically’ modified mice exhibited less huddling contact during sleeping, decreased locomotor activity in novel environments and in learning and memory deficits. These results are consistent with the cognitive impairment and memory loss seen as the earliest clinical symptoms in neurodegenerative disorders such as Alzheimer’s disease. The ApoE knockout mice might therefore be an appropriate model for studying the underlying mechanisms involved in behavioral changes caused by neurodegenerative diseases as well as for evaluating new therapies for these pathologies.

## 1. Introduction

Apolipoprotein (Apo) E had been recognized as a critical protein constituent of lipoproteins, with important functions in controlling lipoprotein metabolism and cholesterol homeostasis. This protein participates in the transport of plasma lipids and in the redistribution of lipids among cells [1]. 

Emerging study has shown that ApoE and ApoE isoform functions may extend beyond lipid metabolism, to include maintenance of normal brain function [2]. Apolipoprotein E is expressed in the central and peripheral nervous systems [3]; produced and secreted mainly by astrocytes [4], ApoE is a major carrier of cholesterols that are required for neuronal activity and injury repair in the brain, so it is probably involved in the removal of debris from damaged cells and in the stimulation of nerve cell regeneration [5].

The knockout mouse model, B6.129P2-Apoe^tm1Unc^ is homozygotic for the Apolipoprotein E (ApoE) deletion; thus, it provides an important tool for studies of the phenotypic consequences of ApoE deficiency. These animals show a marked increase in total plasma cholesterol levels similar to familial type III hyperlipoproteinemia [6].This model was created with the aim of increasing the understanding of the role of ApoE in lipid metabolism, atherogenesis, nerve injury and other biological phenomena [7]. ApoE knockout (ApoE KO) mice are known to mimic some characteristics of the AD clinical phenotype including synaptic damage. However, the biological mechanism involved in this phenomenon is still unclear.

Recent studies in ApoE^−/−^ mice further suggested that ApoE helps to protect the brain against acute injury [8] and maintain neuronal integrity during aging [9]. This idea is supported by the fact that ApoE KO mice exhibit age-dependent synaptic loss and learning deficit [9,10,11,12,13]. Some studies have suggested that ApoE deficiency increases the susceptibility of neurons to oxidative damage [14,15], while others have suggested that loss of ApoE causes impaired blood–brain barrier (BBB) function [16]. Additionally, ApoE knock-out mice exhibit cholinergic dysfunction, tau hyperphosphorylation [11,17] and memory deficits in complex task of hippocampal function [11,18]which are key findings in AD. Finally, cholesterol supply to neurons via ApoE-containing lipoproteins is an important stimulant of synaptogenesis and cholesterol depletion may limit synapse development [19]. Thus, the above-mentioned studies suggest that apoE plays a neurotrophic and neuroprotective function within the CNS and that its deficit could result in neurodegeneration.

However, several results have failed to observe these features particularly in behavioral tests. In fact, some reports have shown contradictory results in which good performances of ApoE^−/−^ mice in spatial tasks have been observed [20,21,22]. These discrepancies could be related to environmental factors, compensation by other proteins, genetic background as well as the protocol and behavioral task employed [23,24]. Thus, due to these conflicting results, additional studies with rigorous analyses of the behavioral phenotypes are still needed.

Consequently, the main objective of this study was to determine the changes related to age and spontaneous behavior and the learning and memory tasks of ApoE KO mice. Since these mice are exposed to hypercholesterolemia from the beginning of early life, our study may help to understand the role of cholesterol metabolism in cognition. The above, may aid to identify an animal model for studying the behavioral changes caused by neurodegenerative diseases as well as for evaluating new therapies to these pathologies.

## 2. Materials and Methods

### 2.1. Animals

Knockout ApoE mice (B6.129P2-Apoe^tm1Unc^/Cenp) and their wild-type control mice (C57Bl/6/Cenp) were obtained from the National Center for Laboratory Animal Breeding (CENPALAB, Havana, Cuba). The animals were housed in 1264C Eurostandard Type II plastic cages (Tecniplast, Italy) with a minimum floor area of 96 cm^2^/animal (range 3–5 animal/cage), grouped by age, sex and genotype. Animals were maintained under controlled temperature (22 ± 2 °C), 60–80% relative humidity, 12-h light/dark cycle and a room air exchange of 12–18 times/h. Animals were given Certified Rodent Diet EMO1004 (ALYco, CENPALAB, Havana, Cuba) in granulated form. Feed and water were sterilized by autoclaving and were available *ad libitum*. Autoclaving was performed at 120 °C for 60 min for water and at 120 °C for 20 min for feed. All animal studies were conducted under a protocol approved by the Institutional Animal Care and Use Committee.

Mice of both strains, aged 5 to 6 months, 12 to 14 months and 18 to 20 months from separate cohorts, were evaluated. Both sexes were used for behavioral testing; female mice were used for learning and memory tests.

### 2.2. Behavioral Tests

The behavioral tests were conducted as previously described to investigate the behavioral phenotypes of transgenic and knockout mice [25]. All mice were examined in order to detect any abnormal physical features, i.e. poorly groomed fur, bald patches in the coat, or an absence of whiskers, which may indicate unusual home cage behaviors [26].

#### 2.2.1. Gross Neurologic Function

Gross neurologic function was evaluated in ApoE^−/−^ and C57Bl/6 mice for detecting severe neurological dysfunction. Mice of both strains aged 5 to 6 months and 12 to 14 months (n = 10/sex/age/genotype) were evaluated. Each mouse was placed in an empty cage for 3 min and presences of abnormal spontaneous behaviors: (wild-running, excessive grooming, freezing and hunched body posture when walking) were recorded.

The sleeping pattern of mice in cages was determined by recording the sleeping positions of mice in their home cages twice daily over 5 consecutive days with the objective to detect abnormal social behavior during the sleeping phase. Mice that slept huddled in one quadrant of the cage were recorded and expressed as a percentage using the following formula:

Animal sleeping huddled (%) = [Mice sleeping in one quadrant/Animals in the cage] × 100

Motor coordination and balance were tested using the accelerating rotarod test (CENPALAB, Havana, Cuba) according to Schumm et al., 2012 [27] with slight modification. Mice were placed on a beam rotating at 9 rpm increasing the speed to 18 and 36 revolutions per minute, over a 5-min period. The time to fall was automatically recorded. Six trials were performed, with a 5-min rest period between trials. The performance on the rotarod was calculated as the mean of the six trials. 

#### 2.2.2. Specific Behavioral Test

The open field test is one of the most common tests used to observe general motor activity because this provide a good measurement of the approach response toward novelty [28]. The apparatus used was a transparent square cage (42 × 42 × 30 cm) (CENPALAB, Havana, Cuba). Each mouse was placed individually in the open field apparatus; vertical activity (steepness) and visited corners during 2 min were recorded as the measure of activity. Mice (n = 10/sex/age/genotype) of both sexes and strains aged 5 to 6 months and 12 to 14 months were evaluated.

### 2.3. Learning and Memory Tests

#### 2.3.1. Forced Alternation Test (Y-Maze)

Forced alternation tests [29] were conducted using a symmetrical Y-maze (BIOCEN, Havana, Cuba). Each arm of the Y-maze was 35 cm long, 5 cm wide and 10 cm high. Mice were habituated to the testing room for at least 30 min. Females C57Bl/6 (n = 8) and ApoE^−/−^ (n = 6) mice aged 18 to 20 months were evaluated. Following habituation, mice were placed in the starting arm and allowed to explore two arms of the Y-maze for 10 min, while the entrance to the third arm was blocked. After the sample trial, the mouse was returned to its home cage for a 30 min inter-trial interval. Later, the block in arm 3 was removed; the mouse was again placed into the start arm and then allowed to access all three arms of the maze for five minutes. Mice were required to enter an arm with all four paws in order for it to be counted as an entry. The time spent in the novel arm was calculated as a percentage of the total time in all three arms during the 5-min retrieval trial. The maze was cleaned with 70% ethanol between the trials.

#### 2.3.2. Novel Object Recognition Test

The novel object recognition test is used to assess recognition memory in mice and is based on the inherent tendency of rodents to explore a novel object longer than a familiar one [30]. Object recognition was developed in ApoE^−/−^ and C57Bl/6 female mice with 18 to 20 months of age (n = 6/genotype). A white-painted wooden pyramid (familiar object, FO) and a white-painted hemisphere (novel object, NO) were used. An acrylic box of 22 × 22 × 25 cm was used. During the habituation phase the animals explored for 15 min (day 1) the empty open-field arena. After 24-h, in the acquisition phase, the mice then explored two identical objects during 7 min. After 1h one of the objects was replaced by a novel and the animal was allowed to explore both objects for 7 min. Behavior was recorded with a non-professional digital camera and the videos were analyzed by a trained experimenter and a stopwatch was used to calculate the time exploring the familiar (TFO) and the novel object (TNO). Exploration was defined if the animal bit the object, touched it with the front extremities or direct the nose at a distance of 2 cm or smaller whereas when the animal pushes the object, sits on it, or approaches the object without paying attention was not considered as an exploration. The Preference Index (PI) representing the percent time investigating the novel object relative to the total object investigation and was used as the main index of retention. This was calculated according to the following formula: PI (%) = [TNO/(TNO+TFO)] × 100 [30].

#### 2.3.3. Passive Avoidance (PA) Conditioning

ApoE^−/−^ (n = 10) and C57Bl/6 (n = 6) female mice with 18 to 20 months of age were evaluated. A step-through PA box was constructed of plastic and had a solid, stainless-steel floor with a 1-cm gap in the middle wired to receive a shock with a homemade grid floor shocker. The apparatus consisted of two compartments, an illuminated compartment (7.5 × 9.3 × 15 cm, with a 60 W light suspended 20 cm above the top of chamber) and a dark compartment (12.5 × 14 × 16.5 cm). The compartments were separated by a guillotine door (3 × 3 cm). For the experiment, all mice were brought to the testing room at least one day prior to testing and the lights in the room were attenuated to allow the animals to acclimate to the darkened room. The day of the experiment, the table lamp over the start chamber was illuminated and then each mouse was picked up gently and placed onto the grid floor in the illuminated area of the box, facing away from the guillotine door. The timer was initiated immediately after the mouse was placed into the chamber. After 5 s, the door to the darkened area of the chamber was opened. The time taken by the mouse to enter the box with all four feet (step-through latency) was recorded. When the mouse entered the box, the opening door was manually closed and the animal received a 50-Hz scrambled shock of 0.5 mA through the wired grid. On the retention test the mouse was placed on the platform as in the training session and the step-through latency was recorded. A maximum of 5 min was given to each animal to enter the box on the testing day. If a mouse failed to walk into the dark chamber within 5 min, maximum 300-s latency was recorded and the mouse was returned to its home cage. The time interval between learning and testing trial was 24, 48 and 96 h for each group.

### 2.4. Statistical Analysis

All statistical analyses were carried out using SPSS Data Analysis Program version 10.0 (SPSS, Inc., Chicago, IL, USA). Statistical evaluation was performed by a randomized complete analysis of variance (ANOVA) or Student *t*-test design with significance assessed at *p* < 0.05 level. When data did not have a normal distribution, the Kruskall–Wallis test and the two-tailed Mann Whitney test were used.

## 3. Results

### 3.1. Gross Neurologic Function

The observation of ApoE^−/−^ and C57Bl/6 mice evidenced the absence of abnormal spontaneous behaviors, such as wild-running, excessive grooming, freezing and hunched body posture when walking. In all animals, the locomotion was correct and no abnormal movements were seen.

**Figure 1:**
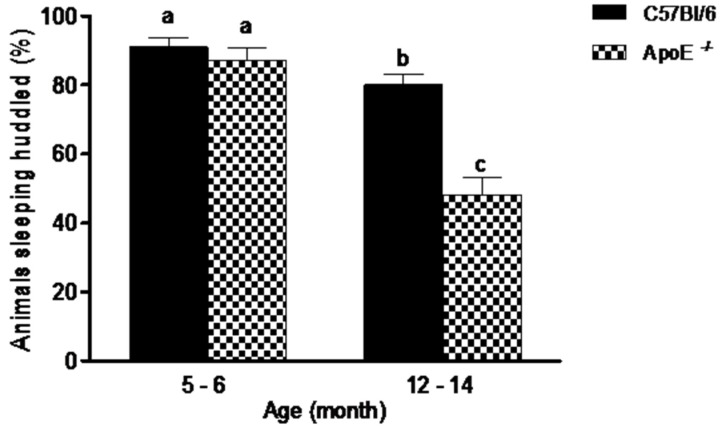
Percent of aged ApoE^−/−^ mice sleeping huddled in the same quadrant decreased significantly in relation to young animals while no differences were detected in C57Bl/6 mice. Sleeping pattern of C57Bl/6 and ApoE^−/−^ mice with different age and sex (n = 10/sex/age/genotype) was evaluated twice daily over 5 consecutive days. Values are expressed as combined male and female data means ± SEM. Different letters (a,b,c) represent significant differences between groups (F(3/39) = 27.15, *p* = 0.000). Kruskal Wallis non-parametric one-way ANOVA and Tukey’s Multiple Comparison Tests. No differences were observed between sexes in both strains.

Social aggregation during sleep test (Figure 1) evidenced no significant differences (*p* = 0.395) between younger mice of both strains. In contrast, in C57Bl/6 mice, the older animals slept huddled in one quadrant of the cage significantly less often than young mice ofthe same strains. There was also, significant differences (*p* = 0.0007) in aged ApoE^−/−^ mice compared to aged-matched C57Bl/6 mice (48% ± 16.2 vs. 80% ± 9.47, respectively), with a decrease in animals observed sleeping near each other. Additionally, a significant difference was observed when compared young and aged C57Bl/6 mice, as well as ApoE^−/−^of the two ages. No differences were founded between sexes in both strains.

**Figure 2:**
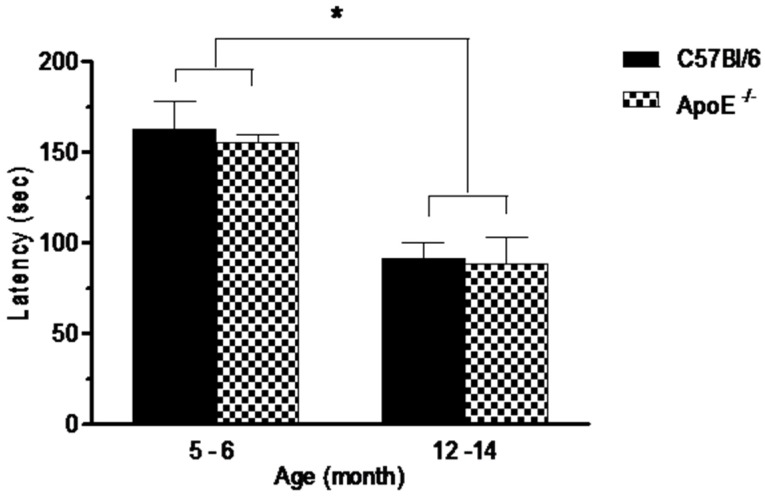
Performances in the rotarod test, expressed as latency to fall off a rotating rod (s) is significantly reduced in aged mice (12 to 14 months) in comparison with younger mice (5 to 6 month). No differences were seen between strains or sexes (n = 10/sex/age/genotype). Values are expressed as combined male and female data means ± SEM. * Groups with statistically significant differences (F(3/54) = 14.17, *p* = 0.000). Kruskal Wallis non-parametric one-way ANOVA and Tukey’s Multiple Comparison Tests.

Performances on the rotarod test are shown in Figure 2. Statistical analysis showed a significant decrease (*p* = 0.0073) on the average latency in the time remained in the apparatus in older mice compared to younger mice in both strains (91.2 ± 20.4 and 88.3 ± 53.7 vs. 162.6 ± 40.0 and 154.8 ± 26.4, respectively) although no differences (*p* > 0.05) were detected between knockout and wild type mice at the same age (Figure 2) or between sexes in both strains.As rotarod involves mainly motor coordination, balance and muscular strength [31] our results suggest that Apolipoprotein E annulation does not induce motor dysfunction.

### 3.2. Specific Behavioral Test

**Figure 3:**
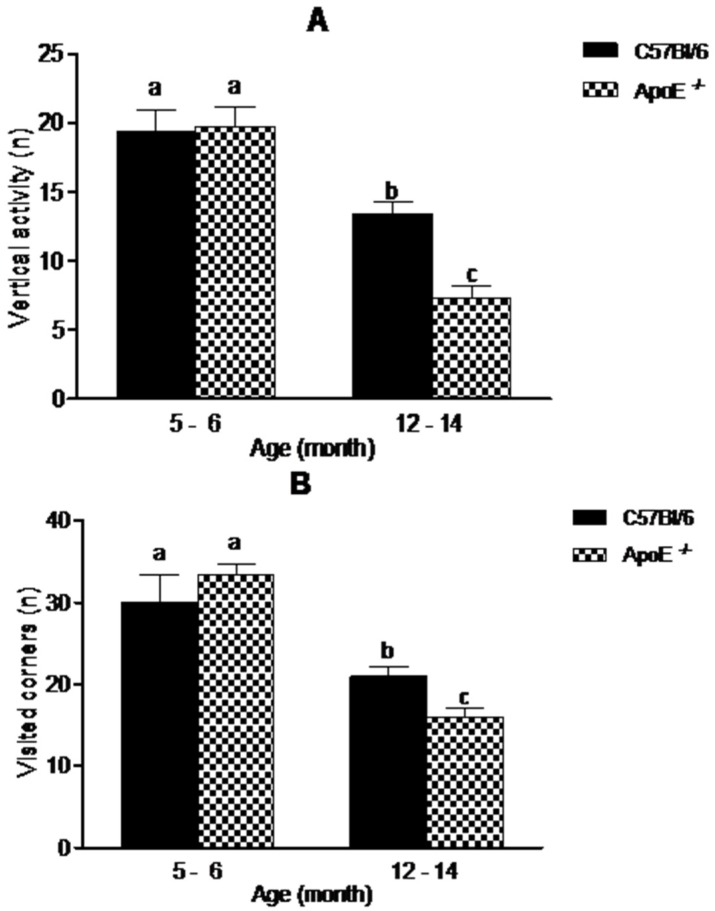
Reduction in exploratory behavior in open field is associated with aging in C57Bl/6 and ApoE^−/−^ mice (n = 10/sex/age/genotype) although knockout mice showed significantly decrease in exploratory activity compared with their age-matched wild-type controls. Values are expressed as combined male and female data means ± SEM. Different letters (a,b,c) represent significant difference between groups. (**A**) Vertical activity (steepness) (F(3/34) = 21.38, *p* = 0.000); (**B**) Visited corners. (F(3/23) = 16.38, *p* = 0.000). Kruskal Wallis nonparametric one-way ANOVA and Tukey’s Multiple Comparison Tests. No significant differences were found between sexes in both strains.

The open field test (OFT) is one of the most common tests used to measure exploratory behavior and general activity [28]. In our study (Figure 3), aging was associated with a decreased exploratory activity. There was a significant decrease in vertical activity (19.09 ± 4.61 vs. 13.38 ± 2.45 in C57Bl/6; 19.75 ± 3.92 vs. 7.25 ± 2.82 in ApoE^−/−^) and visited corners (30.00 ± 8.29 vs. 21.00 ± 2.76 in C57Bl/6; 33.33 ± 3.27 vs. 16.00 ± 2.53 in ApoE^−/−^), (*p* < 0.05). Interestingly aged ApoE^−/−^ mice exhibited significant decrease compared to C57Bl/6 aged mice, although no significant differences were observed between sexes in both strains. 

### 3.3. Learning and Memory Tests

#### 3.3.1. Forced Alternation Test (Y-Maze)

**Figure 4:**
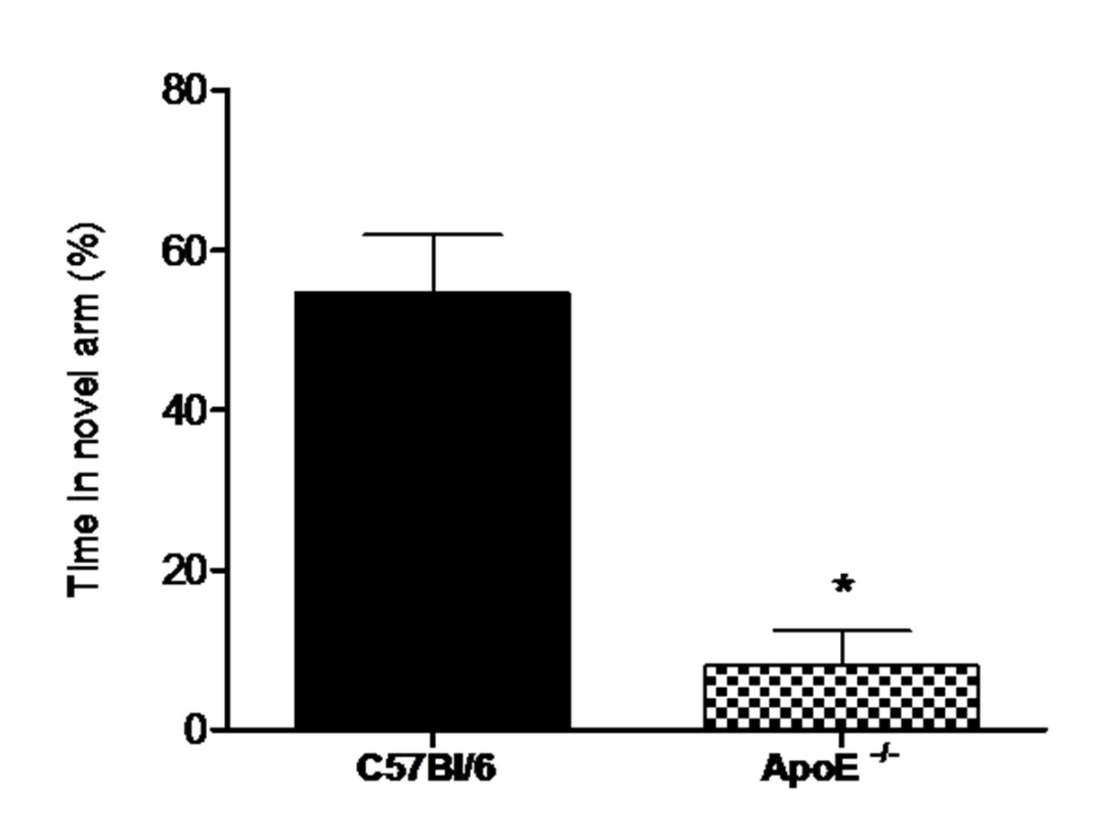
Female ApoE^−/−^ mice (18 to 20 months) (n = 6) spent significantly less time in the novel arm compared to age-matches C57Bl/6 mice (n = 8). Values are expressed as means ± SEM. * Significant difference Vs C57BL/6 mice (T(7) = 5.60, *p* = 0.0018). Mann Whitney U Test.

Alternation tasks measure the disposition of rodents to explore new environments and are used to evaluate working memory and exploratory behavior [32].Our results showed (Figure 4) that the percent time spent in the novel arm was significantly higher for female wild type mice compared to knockout mice (58.8% ± 7.26 vs. 8% ± 4.38, *p* = 0.02).

#### 3.3.2. Novel Object Recognition Test

**Figure 5:**
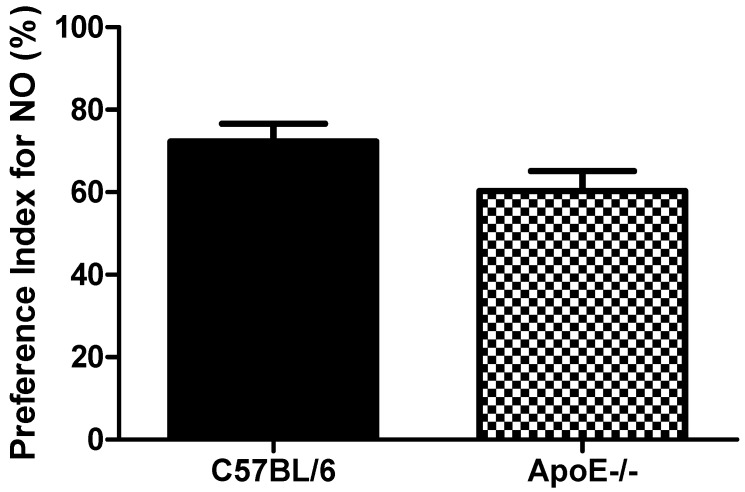
Preference Index in female C57Bl/6 and ApoE^−/−^ mice (18 to 20 months) Values are expressed as means ± SEM (n = 6). No significant differences between groups (T(10) = 1.87, *p* = 0.091). Mann Whitney U Test.

Object recognition test showed(Figure 5) that aged C57Bl/6 mice tended to increased PI when compared to ApoE^−/−^ mice (72.3 ± 10.5 vs. 60.3 ± 11.8, respectively, *p* = 0.091). These results suggest that object recognition is not significantly impaired in 18 to 20-month old knockout mice when compared with age-matched control mice (Figure 5). The relative inability of aged ApoE KO mice to master the task may reflect a diminished ability for short term memory retention. However, the low number of mice (n = 6) in each group may have contributed to the lack of significance found for this cognitive paradigm.

#### 3.3.3. Passive Avoidance (PA) Conditioning

**Figure 6:**
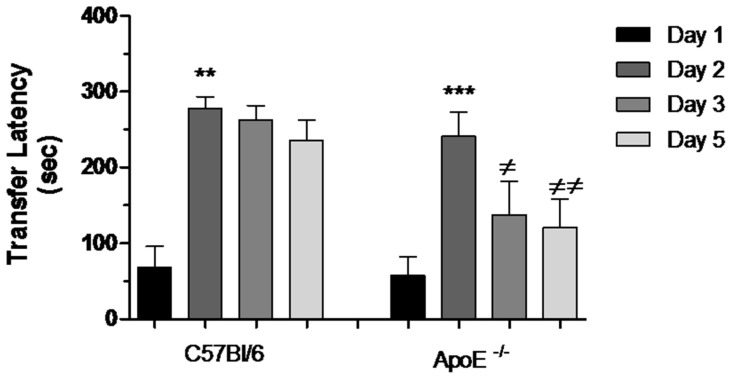
Reduction of transfer latency between Days 2, 3 and 5 in female ApoE^−/−^ mice. Transfer latency to entering the dark compartment was measured on Days 1, 2, 3 and 5 in C57Bl/6 and ApoE^−/−^ mice. Values are expressed as means ± SEM. Transfer latency increased significantly on Day 2 in both strains: C57Bl/6 (n = 6) and ApoE^−/−^ mice (n = 10) (** *p* < 0.01 and *** *p* < 0.001, respectively, Mann Whitney Test). However, significant difference was found between Days 2, 3 and 5 exclusively in ApoE^−/−^ mice (≠ *p* < 0.05; ≠≠ *p* < 0.001, F(2/29) = 6.236, *p* = 0.0005. Repeated measured ANOVA and Bonferroni’s Multiple Comparison Test).

During the acquisition session of the task, all mice readily entered the dark compartment and had similar approach behaviors toward this compartment. There was no difference in the latencies to enter the dark compartment between old C57Bl/6 mice (68.00 ± 298.09) and ApoE^−/−^ mice (58.30 ± 23.40). During the retention session, 24 h later, the groups did not differ in terms of the latency to enter the dark compartment (277.50 ± 15.55 vs. 241.10 ± 32.48). Therefore, there was a significant learning after acquisition test (Day 1). Nevertheless after 48 h, a significant impairment was evidenced in ApoE^−/−^ mice, characterized by reduction in transfer latency between Days 2, 3 and 5 (Figure 6).

## 4. Discussion

Cholesterol homeostasis is important for normal brain functions, since it is an essential component for axonal growth, synaptic formation and remodeling events that are crucial for learning and memory [33,34]. Cholesterol dysfunction in the CNS could be associated with aging and the development of certain neurodegenerative diseases [19,35].

B6.129P2-Apoe^tm1Unc^ mouse is a knockout mouse model, homozygotic for the mutation Apo-E^tm1Unc^; thus, it develops dysfunction of cholesterol metabolism characterized by hyperlipidemia and atherosclerosis [7]. The study of this animal as model of neurodegenerative diseases indicates an exciting potential for studying the underlying mechanisms involved in behavioral changes with general applicability to many of these diseases.

Several results have showed ApoE deficient mice with cognitive impairments. It has been shown that ApoE^−/−^ mice exhibit learning deficit in behavioral tasks related to hippocampal functions [9,11,12,36]. However, results of experiments in water maze are conflicting and to some extent difficult to interpret despite these mice show altered long term potentiation [9,37] as well as dysfunction of the cholinergic system [36]. Besides, regarding neurological function and emotional task, some studies have shown no aged-related impairment of balance, strength, or coordination [20,21,22].

The disagreement in some neurological abnormalities may support that environmental variables, housing and management as well as methodological approaches must be standardized to avoid potential differences between distinct groups of ApoE^−/−^ mice [10]. To overcome some of the limitations of previous studies, we developed not only memory and learning tasks but rather a general neurologic function battery including social aggregation during sleep test, motor activity measured by rotarod test as well as exploratory behavior. These tests were used to gain further description of the aging process in ApoE^−/−^ mice. We thought this approach of particularly interest since this animal model could allow us to learn more about physiopathological pathways that underline hypercholesterolemia and dementia.

The experiments reported in the present study in relation with gross neurological function and exploratory activity involved mice of both sexes aged up to 12 months. This age was selected as these mice consistently showed significant age-related behavior changes [38,39]. Normally, aging is considered to be associated with progressive changes in the brain and it is associated to the impairment of sensory and motor function. Interestingly, our study showed that aged ApoE^−/−^ mice displayed striking abnormalities in social behaviors, exploratory activity compared to age-matched wild type strain C57Bl/6. However, motor activity was apparently normal. Therefore, despite other studies have not detected age-related neurological abnormalities in ApoE^−/−^ mice [20,40] our result strongly suggests that hypercholesterolemia during aging is related with these changes.

We next determined whether 18 to 20 month mice showed evidence deficits in learning and memory because we were interested in detecting memory impairment in older mice. Relative to the maximum life span, this age may be considered to represent an old mouse for setting up experimental murine models more analogous to senescent humans [41]. Our results of learning and memory tests were obtained with aged virgin female mice. A general approach is to use male mice to avoid potential behavioral variability due to the estrous cycle. However, previous studies in transgenic mice including ApoE^−/−^, evidenced increased susceptibility of females to cognitive dysfunction [42,43]. Thus, considering that the risk of AD is higher in women than in men [44,45], the selection of female mice underscores the significance of this model. Furthermore, male mice could be aggressive and inflict considerable damage each other over a long period of time that made female mice more suitable for very long-term studies.

In our experiments, we tested whether there would be differences between aged ApoE^−/−^ female mice and age-matched wild type strain in emotional learning and memory using the passive avoidance test. One first interpretation of our results may assume that the deficit in learning of ApoE^−/−^ mice may be caused by their poorer acquisition. However, this seems unlikely considering that there was no difference in passive avoidance performance in memory retention 24 h (Day 2). Therefore, another possibility involves decreased retention of the task as an increased difference in performance was observed while the time between training and testing progressed. Hence, in our opinion the most striking interpretation is that this deficit may reflect impairment related to the inability of ApoE^−/−^ mice to store or retrieve the recent information in memory. Our results are in agreement with the neurodegenerative changes [9] and the cholinergic deficit [36] previously observed in these mice and with the sensitivity of this test to cholinergic manipulations [46,47].

The novel object recognition test has been developed to study learning and memory in rodents. The brain areas involved in this test are mainly hippocampus and perirhinal cortex [30]. Therefore, our results neither correlated well with the neurodegenerative changes in hippocampus and cortex previously observed in ApoE deficient mice nor with the behavior of mice with central cholinergic dysfunction in the novel object recognition test [23,30,47]. Taking into account our previous results in passive avoidance test, we certainly believed that relative inability of aged ApoE KO mice to master the task could reflect a diminished ability for short term memory retention, however the low number of mice (n = 6) in each group may have contributed to the lack of statistical significance found for this test. Accordingly, new experiments are necessary in order to corroborate this hypothesis.

In humans ApoE is the major cholesterol carrier for lipid transport. Several findings suggest that ApoE polymorphic alleles are the main genetic factor for AD. Evidence has suggested that individuals carrying the ε4 allele are at higher risk compared with that carrying ε3 allele, while the ε2 allele decreases risk [48,49,50]. At present, it is known that ApoE isoforms differentially regulate Aβ aggregation and clearance in the brain and have distinct functions in regulating several other pathologies involved in AD including diabetes [50]. ApoE ε4 has been linked to an increase Aβ accumulation, aggregation and deposition in the brain and for instance, this allele is associated with enhanced amyloid pathology even in cognitively normal people [51]. Also, people that have both diabetes and an ApoE4 allele increase are prone to develop AD by increasing the amyloid deposition [52].

However, mice express only one type of ApoE [53] and, therefore, the usefulness of this model to extrapolate their results with what occurs in humans is still debatable. In fact, it is noteworthy to note that there is evidence that mouse and human ApoE have differing biological effects that may involve some clearance mechanisms. In this regard, it should be pointed out that mice have substantially different relative lipoprotein levels from humans and they use HDL as a major cholesterol carrier [54], while humans use LDL. Thus, the immediate phenotype of ApoE deficiency may be different in mice from that in humans.

It is noteworthy that ApoEknock-out mice have highly increased plasma lipid levels [55,56], which may independently cause synaptic dysfunction and cognitive deficits [57]. Hypertriglyceridemia in mice causes cognitive dysfunction [58] and numerous studies have investigated the effect of high-fat diets on cognition in animal and human models [59]. This is consistent with a recent reported study, where genetic restoration of plasma ApoE to wild-type levels normalized plasma lipids in ApoE KO mice. While this did not rescue synaptic loss, it does completely restore learning and memory in the mice, suggesting that both CNS and plasma ApoE are independent parameters that affect brain health [43]. One mechanism by which ApoE loss could lead to cognitive impairment is its effect on the vasculature. Vascular dysfunction and atherosclerosis occur early in ApoE KO mice, which leads to reduced cerebral blood flow and dysfunctional autonomic regulation of the cerebrovasculature [55,56,60]. In addition to vascular changes, ApoE knock-out mice exhibit an age-dependent breakdown of the BBB, which correlates with behavioral impairments [61,62].

Growing evidence suggests that hypercholesterolemia during adulthood is associated with a large increase in the prevalence and incidence vascular dementia and AD [63,64]. Therefore, as ApoE deficient mice are exposed to hypercholesterolemia from the beginning of early life, this model is potentially relevant to Alzheimer’s disease (AD). Thus, the ApoE knock out mouse model should help us to understanding the underlying biological mechanisms of neurodegenerative disease and the role of cholesterol metabolism in cognition. Our current data may help to provide a suitable animal model with potential application in neurobiological sciences, however, a more complete biochemical and behavioral characterization of this model is needed.
